# The Case for Early Palliative Care in the Treatment of Ovarian Cancer

**Published:** 2014-07-01

**Authors:** Lauren Hardiman

**Affiliations:** From University of South Florida–College of Nursing, Tampa, Florida

Once referred to as a silent killer but more recently as "the disease that whispers," ovarian cancer’s early-stage symptoms are often vague and mimic other gastrointestinal or genitourinary illnesses. Although the median age at time of diagnosis is 63 years old, most women attribute the nonspecific nature of symptoms to menopause or stress (National Cancer Institute [NCI], 2014). Over time, tumor growth produces the hallmark clinical findings of pelvic ascites, abdominal distension, and pain, leading the patient to seek out evaluation and eventual diagnosis and treatment. The majority of women (61%) who present for evaluation will already be in the advanced stages of the disease (NCI, 2014).

According to the NCI, there will be an estimated 22,240 new cases of ovarian cancer in the United States this year, as well as a resultant 14,030 deaths (NCI, 2014). It is the deadliest of all gynecologic cancers. Though this disease accounts for only 3% of all cancer cases in women, it is the fifth leading cause of cancer-related death (Martin, 2011; NCI, 2014). There has been no improvement in the mortality rate in the past 40 years. Advanced ovarian cancer runs a chronic course, often with variable phases of latency. In three quarters of patients, tumor recurrence will occur. Long-term survival of greater than 5 years for those with advanced disease will only be achieved in approximately 25% of patients (NCI, 2014; Ozols, 2005).

## Treatment

The primary intervention for patients with ovarian cancer is complete/optimal surgical cryoreduction. In the case of advanced disease, a debulking surgery will be performed involving a total hysterectomy; salpingo-oophorectomy; omentectomy; lymph node sampling; and the removal of as much tumor as possible, which may include resection of the peritoneum, diaphragm, and areas of the bowel (Martin, 2011).

Surgery is frequently followed by the administration of combination chemotherapy using paclitaxel plus carboplatin. For some patients, intraperitoneal chemotherapy with cisplatin will be used to improve progression-free and overall survival (Martin, 2011). Most women will eventually become resistant to first-line therapies and will require treatment with second-, third-, and possibly fourth-line chemotherapy regimens.

## Symptom Burden

Symptom management of patients with ovarian cancer is done along a continuum: It begins at diagnosis and continues to the end of life. Symptoms can be both disease- and treatment-related. The most common symptom experienced from diagnosis through completion of treatment is fatigue (Donovan, Hartenbach, & Method, 2005; Ferrell, Smith, Cullinane, & Melancon, 2003; Price et al., 2013). Abdominal and pelvic pain is associated with both treatment and the disease itself. Disease- and treatment-related gastrointestinal symptoms are frequent. Symptoms include bloating, nausea and vomiting, constipation, anorexia, and diarrhea (Ferrell et al., 2003; Herrinton et al., 2007). In advanced disease, bowel obstructions can occur due to compression of the tumor on the intestine or decreased peristalsis. In addition, when patients become resistant to chemotherapy, ascites can return. Chemotherapy can result in hair loss, neutropenia, and thrombocytopenia, as well as peripheral neuropathy and memory problems.

## Early Palliative Care: Symptom Management Through the Trajectory of Disease

Optimal symptom management in advanced ovarian cancer can be achieved by integrating early palliative care with early cancer care. According to the World Health Organization (WHO), palliative care "is applicable early in the course of illness, in conjunction with other therapies that are intended to prolong life, such as chemotherapy or radiation therapy, and includes those investigations needed to better understand and manage distressing clinical complications" (WHO, 2014). Comprehensive treatment that includes surgical, radiation, and medical oncologists in collaboration with palliative care allows the oncologist to focus on the management of cancer while the palliative care team addresses a large number of physical and psychosocial concerns (Bruera & Hui, 2010). Co-management of ovarian cancer patients allows enhancements to quality of life by relieving symptoms associated with cancer and its treatment effects (Meier & Brawley, 2011).

## Role of Palliative Care

The scope of palliative care varies across sites and locations and ranges from only end-of-life care to the management of symptoms and other distressing issues involved throughout the trajectory of disease. Recent definitions of palliative care (see Figure) describe a comprehensive interdisciplinary approach focused not only on symptom management but also on the physical, psychosocial, and spiritual needs of patients and their families during a serious illness (Center to Advance Palliative Care, 2011; Levy et al., 2012; Smith et al., 2012; WHO, 2014). Palliative care specialists/teams seek to provide an additional layer of support and education beyond that of the primary team. They work with both patients and their caregivers. Care is often delivered as a holistic, empathetic resource for communication regarding disease, prognosis, and disease-related treatment choices in addition to management of intolerable symptoms and psychosocial issues.

**Figure 1:**
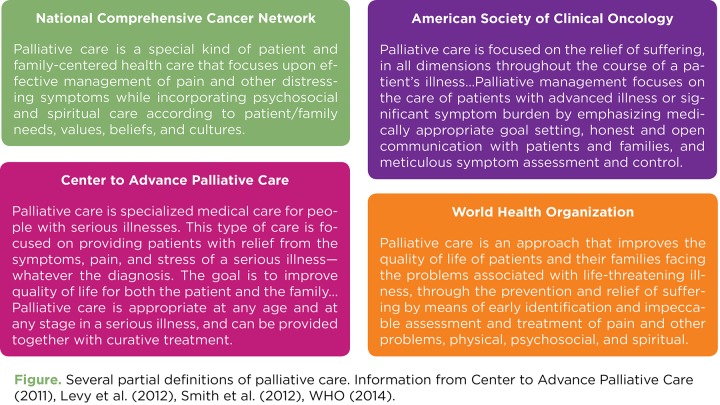
Several partial definitions of palliative care. Information from Center to Advance Palliative Care (2011), Levy et al. (2012), Smith et al. (2012), WHO (2014).

## Evidence for the Early Integration of Palliative Care

One of the most widely received studies on the early integration of palliative care was performed on a population of patients newly diagnosed with metastatic non–small cell lung cancer. Patients with metastatic non–small cell lung cancer, like those with ovarian cancer, have a substantial symptom burden and may receive aggressive care at the end of life. Their prognosis is often grim.

In a randomized controlled trial, Temel and colleagues investigated the impact of early palliative care on quality of life and survival (Temel et al., 2010). They found that patients who received early palliative care experienced significant improvement in quality of life compared with patients who received standard care. Patients who participated in the intervention also reported lower rates of depression and were more likely to have their resuscitation preferences documented in the medical record (Temel et al., 2010). In addition, after the study had concluded, analysis demonstrated that those who received early integrated palliative care had a longer median survival than those in the standard-care group.

Bakitas and colleagues (2009) carried out Project ENABLE (Educate, Nurture, Advise, Before Life Ends). This randomized controlled trial assigned individuals who were newly diagnosed with advanced gastrointestinal, lung, genitourinary, and breast cancers to receive a multicomponent nursing-led intervention in collaboration with standard care vs. standard care alone. For those receiving the intervention, advanced practice nurses with palliative care training conducted formal education sessions with patients, followed by monthly telephone contacts for ongoing case management that included assessments for additional referrals or resources, until the time of the patient’s death (Bakitas et al., 2009). Results demonstrated that patients assigned to the intervention group reported significantly better quality of life and mood, as well as positive effects on symptom intensity. Post-study analysis demonstrated that patients who received the intervention had a greater median survival time, but this difference was not statistically significant.

More recently, a pilot randomized trial sought to evaluate the benefits of a nurse practitioner–directed palliative care intervention for patients with metastatic cancer (Dyar, Lesperance, Shannon, Sloan, & Colon-Otero, 2012). Patients who received the intervention had an initial consultation (and a 1-month follow-up) with an oncology nurse practitioner who taught them about hospice; helped them to fill out living will forms; and also assessed their psychological, physical, cognitive, social, and spiritual needs. The intervention group showed statistically significant improvement in emotional and mental well-being, as well as statistically improved quality of life, compared with the control group. This study was limited in that it had a small cohort of only 26 patients. The results demonstrated by the outcome improvements related to the nurse-driven intervention, as well as patient feedback, encouraged widespread adoption of the intervention (Dyar et al., 2012). For ethical reasons, researchers decided to forego accruing greater participation into the control population.

## Barriers to Care

Confusion regarding the term "palliative care" often leads to underutilization or delayed referrals until late in the disease trajectory. Physicians often equate palliative care with end-of-life care or hospice and have feelings that referral to palliative care will destroy hope (Hui et al., 2012). Some see palliative care as simply "the pain team." Both physicians and patients must come to understand that the inclusion of palliative care into treatment should not be considered giving up on the patient, but rather sharing expertise between both teams to continue disease-modifying therapy while optimizing patient and caregiver quality of life and outcomes.

## Conclusion

In the context of advanced disease, quality of life is most affected by the symptoms patients experience. Ovarian cancer, which most often presents in the later stages of disease, carries a heavy symptom burden related to both the disease process and its treatment interventions. Recent studies have exhibited the positive impact of early palliative care on non–small cell lung cancer patients who, like those with ovarian cancer, carry a high burden of disease and poor prognostic outcome. Early integration of palliative care into the collaborative management of ovarian cancer patients may lead to improved patient outcomes and greater quality of life throughout the disease trajectory. Palliative care is not restricted to those who are at the end of life but rather is aimed at allowing patients and their caregivers to experience quality of life without the burden of distressing symptoms and anxieties related to illness. 
